# Wessex Branch of the Association of Clinical Pathologists

**Published:** 1992-03

**Authors:** 


### Wessex Branch of the Association of Clinical Pathologists

**The Winter meeting of the Wessex Branch of the Association of Clinical Pathologists was held at the Postgraduate Medical Centre, Taunton and Somerset Hospital, Musgrove Park, Taunton on Thursday 5 December, 1991. Approximately 40 pathologists from all disciplines attended. Papers, abstracts of which follow, were presented by trainees. The size of the audience at the end of the meeting reflected its success.**

### DETECTION OF HERPES SIMPLEX VIRUS DNA IN THE BRAIN AND CSF BY USE OF THE POLYMERASE CHAIN REACTION

**J. A. R. Nichol, S. Love**
Department of Neuropathology
Frenchay Hospital

Herpes simplex virus type 1 (HSV-1) is the commonest cause of encephalitits in the United Kingdom. We have been able to detect HSV-1 DNA, by use of the polymerase chain reaction (PCR), in formalin-fixed paraffin-embedded tissue from the brains of 6 patients who died during the acute phase of the illness. In all 6 cases viral antigen was readily demonstrated by immunohistochemistry. We have also examined the brains of 3 patients who died several years after an acute encephalitis, the cause of which had not been diagnosed during life or at post mortem. Despite the absence of detactable viral antigen, HSV-1 DNA was amplified from all 3 brains suggesting a persistent low grade or possibly latent infection. No HSV-1 DNA was detected in control brains.

PCR-mediated demonstration of viral DNA in CSF has been shown to be a sensitive and specific means of diagnosing herpes simplex encephalitis during life. We have examined the CSF of 7 patients with a clinical diagnosis of possible herpes encephalitis and amplified HSV-1 DNA from only one. In this case the clinical diagnosis was supported by the findings of temporal lobe lesions on a CT scan and a temporal lobe focus on EEG. Alternative diagnoses were subsequently made in 2 of the 6 patients without demonstrable viral DNA.

### A REAL MODEL OF ULCERATIVE COLITIS

**B. F. Warren, P. E. Watkins, A. Terlecki, G. R. Pearson, J. Price, J. W. B. Bradfield and I. A. Silver**
Departments of Pathology and Anatomy
University of Bristol

The cause of human ulcerative colitis is unknown. Medical treatment is not curative. The pathogenesis of its extracolonic manifestations and intracolonic complications is not understood. A model in which to study the disease further is essential. Many chemically induced models of colonic disease have been claimed to resemble ulcerative colitis. They are usually a good model of inflammation but not of ulcerative colitis. Most spontaneously occurring models have been found to be due to an infection. The cotton top tamarin, a New World monkey, develops a spontaneously occurring colitis which resembles human ulcerative colitis clinically, endoscopically, histologically and in its response to treatment. The cottom top tamarin also develops multiple, flat, right sided colon cancer and liver disease.

This paper describes 60 endoscopic examinations in the cotton top tamarin and a classification of the colitis which occurs, and provides justification for its place as the only real model of human ulcerative colitis.

### CAMPYLOBACTOR COLITIS IN TOP TAMARINS

**P. E. Watkins, B. F. Warren**
School of Medical Sciences
University of Bristol

Campylobacter infection has been reported in a range of animals, including primates. Although it is often associated with overt clinical disease, infection has been recorded in normal animals.

Campylobacter jejuni infection has been diagnosed in a colony of Cotton Top Tamarins which also suffer from a form of ulcerative colitis. Clinical signs associated with infection include diarrhoea and dehydration, often with rapid progress of clinical signs.

Diagnosis of infection has been made from culture of fresh faecal samples (often removed directly from the rectum) and, in some cases, by examination of rectal biopsies. The latter demonstrate histological features markedly different from the changes observed in ulcerative colitis.

Treatment has involved oral erythromycin, administered in drinking water. In severe cases, antibiotic therapy has been combined with supportive fluid therapy, plus flunixin to counteract endotoxaemia.

Colony health has been monitored by undertaking regular faecal screening from all cages. Any animal positive to culture has been treated with erythromycin, and subsequently re-cultured.

Within the colony, recurrence of infection is not uncommon. The source of the re-infection is unclear although a carrier state in an animal appears unlikely, based on the results of repeat faecal screens.

### LYMPH NODE SIZE IN COLORECTAL CARCINOMA ACCORDING TO DUKES' STAGE AND 5 YEAR SURVIVAL

**C. P. Case**
Department of Histopathology
Southmead Hospital, Bristol

The presence of a pronounced lymphocytic infiltration around the edge of a rectal carcinoma was originally shown in 1931 by McCarthy to be associated with a favourable prognosis. This has been confirmed by many authors. Recently Jass (1986) has shown that the lymphocytic infiltrate, which is most pronounced is Dukes' A, less in Dukes' B and least pronounced in Dukes' C stage disease, is an independent prognostic variable to survival. Two theories were proposed by Jass to account for this. One that it reflects a specific response by the host against the tumour. The second favoured explanation was that it was closely related to normal lamina propria in terms of structure and function and might therefore reflect a high level of tissue differentiation. More proximally, the reaction of lymph nodes has also been studied but results have been more equivocal. Some authors have suggested that paracortical activity and sinus histiocytosis are associated with a better prognosis, although not with Dukes' stage. Others have failed to confirm this.

In this study, the size of the lymph node has been studied as a measure or overall lymph node activity. The minimum intra capsular diameter of a mean 5.5-7.2 lymph nodes per case has been measured in 40 cases each of Dukes' stage A, B and C adenocarcinomas found in rectum, sigmoid colon and caecum. The results show no difference in either mean or range of diameter of lymph nodes not containing tumour in Dukes' A, B or C cases. Lymph nodes containing tumour (in Dukes' C) were larger but only by 1mm on average. Similar results were found comparing the mean lymph node diameters for each patient, and for carcinomas of rectum, sigmoid colon and caecum. Lymph nodes in the caecum were larger than those in rectum and sigmoid colon.

A minimal increase in size of mean lymph node size was found in survivors at 5 years after operation for Dukes' A and B adenocarcinoma compared to non survivors at a similar Dukes' stage but this was not statistically significant. No difference was seen in lymph nodes of survivors compared to non survivors at Dukes' C stage whether or not they contained tumour metastases.

In conclusion the size of local lymph nodes appears to be unrelated to the proximity or the extent of the tumour mass and offers no prognostic guide to survival. The apparent lack of reaction of lymph nodes to tumour is in keeping with Jass's theory that the lymphocyte infiltration around the tumour is a measure more of tissue differentiation rather than of host response and accords with recent experiments showing only weak cytotoxic activity of tumour infiltrating lymphocytes against tumour cells in tissue culture.

### MORPHOLOGY AND FLOW CYTOMETRY OF RENAL CELL CARCINOMA

**M. R. Novelli, A. G. MacIver***
Department of Pathology and Microbiology
University of Bristol
Department of Histopathology
Southmead Hospital, Bristol*

Histological assessment and DNA flow cytometry were performed on 15 cases of renal cell carcinoma with macroscopic tumour invasion of the renal vein. In each case samples from the main tumour mass were compared to a single sample of invasive tumour lying within the renal vein.

Comparison of morphological variables showed that samples of the intravenous tumour was more commonly composed of granular cells (53%) than the main tumour (16%) and were of higher nuclear grade.

Flow cytometric DNA studies showed more aggressive tumour lying in the renal vein in 7 of 14 cases, despite multiple samples of primary tumour being compared to a single sample of intravenous tumour in each case.

These findings show that both morphological and biological differences exist between invasive tumour cells in the renal vein and those in the main tumour mass.

### THE DETECTION OF MINIMAL RESIDUAL DISEASE IN CHILDHOOD ACUTE LYMPHOBLASTIC LEUKAEMIA

**M. N. Potter[1, 2], C. G. Steward[1, 2], N. J. Maitland[2] and A. Oakhill[1]**
Depts. of Haematology-Oncology[1], Royal Hospital for Sick Children, Bristol and Pathology[2], Bristol University Medical School

Rearrangement of the immunoglobulin heavy chain gene (IgH) generates a hypervariable sequence known as the complementarity determining region-III (CDR-III). This sequence represents a unique clonal marker for B-lineage lymphoproliferative disorders such as the majority of cases of childhood acute lymphoblastic leukaemia (ALL). We have used the polymerase chain reaction (PCR) to amplify the CDR-III in over 50 cases of B-lineage ALL. Direct DNA sequencing of the PCR products from 55 IgH alleles in 36 of these cases has confirmed the unique nature of the CDR-III in each patient's leukaemia clone and shown preferential involvement of certain joining segments (J4, J5, J6) and diversity regions (DLR/DXP). Following sequence analysis, we have synthesised patient-specific oligonucleotide gene probes based on the most variable region of junctional sequence within the CDR-III. In serial dilution experiments of leukaemia DNA into normal control DNA this has allowed the consistent detection of the malignant clone at a level representing 1 cell in $10^4$, an improvement of at least 2 orders of magnitude over light microscopy or a Southern blot approach.

In a sub-group of children we have performed retrospective studies of the detection of minimal residual disease (MRD) by this approach. The following observations of clinical relevance have been made:

i) the detection of occult marrow disease in CNS-based ALL with normal marrow morphology (n=2).
ii) the detection of MRD in autologous marrow harvests (n=4).
iii) the persistence of MRD in cases of ALL subsequently relapsing (n=6).

The discriminitive value of this sensitive technique in predicting future relapse may however be limited by the presence of detectable MRD in most cases of ALL for the first 6-18 months of therapy, and clonal progression with change in the IgH CDR-III between presentation and relapse. We have observed this event in almost 20% of cases studied.

The detection of MRD in childhoold ALL by IgH CDR-III PCR is clearly possible though further research including prospective studies are required before the full clinical significance and possible applications of this new and exciting approach are realised.

### COMPARATIVE ANALYSIS OF THE NATIONAL EPIDEMIOLOGICAL SURVEY OF SELECTED INFECTION DATA FOR THE BRISTOL ROYAL INFIRMARY

**I. J. Hassan, British Council Scholar**
Microbiology Department
Bristol Royal Infirmary

This survey was part of a multi-centre collection of clinically relevant data on infection in 14 centres in the British Isles. One aim was to build up a national picture of the incidence of bacterial species in specified types of infection: bacteraemias, pneumonias and infections of bone and joint, central nervous system, vascular lines and wounds after 'clean' surgery. Each infection was also classified as community- or hospital-acquired.

The BRI contributed 11.6% of the total number of patients, with a mean age of 43.6 years and a male/female ratio of 1:2, similar to the national findings. Bacteraemias accounted for 57.7% of the infections, pneumonias 20.5%, bone and joint 4.3% central nervous system 6.8%, vascular lines 6.4% and 'clean surgical' wounds 3.9%. In general, the BRI incidence figures tallied with the pooled national data. However, we saw more infections by **Pseudomonas aeruginosa** and **Candida** species, and recorded a higher percentage of **Streptococcus penumoniae** bacteraemia (12.0%; national 7.0%). Of the BRI bone and joint infections 20.1% were due to viridans streptococci, reflecting the large number of infections of joint prostheses and recent trends in the bacteriology of these. Among the nationally reported line infections 30.3% were due to **Staphylococcus aureus,** compared with only 8.3% at the BRI, where the expected preponderance of coagulase-negative staphylococci was seen. We also recorded more pneumonias due to **Streptococcus pneumoniae** (46.4%; national 34.9%) as against **Haemophilus influenzae** pneumonias (26.8%; national 40.5%).

### AN EVALUATION OF TESTS USED IN THE DIAGNOSIS OF POLYCYSTIC OVARIES IN WOMEN WITH OLIGO-AMENORRHOEA

**Paul E. Thomas[1], Robert Fox[2], Michael G. R. Hull[2]**
[1]Department of Clinical Chemistry, Southmead Hospital, Bristol and [2]University Department of Obstetrics and Gynaecology, Bristol Maternity Hospital, Bristol

The performance of (1) hormone measurements, (2) an assessment of hirsutism, (3) oestrogen state as defined by progestogen challenge test, was evaluated in the diagnosis of polycystic ovaries (PCO) using ultrasonography as a reference test, in 65 patients with functional oligo-amenorrhoea.

Of the 65 patients studied 48 had PCO. The best tests as shown by overall diagnostic accuracy (sum of correct positive and negative results as a proportion of all cases) were the free androgen index (FAI) 94% and assessment of oestrogen state 89%. These were significantly better than all other tests: oestradiol, 66%; LH, 69%; LH:FSH, ratio 66%; testosterone, 71% and assessment of hirsutism, 52%. The poorer diagnostic accuracy of these tests resulted from their lower sensitivity 35-69%. The best combinations were the FAI and LH (97%) and those incorporating the assessment of oestrogen state (91-92%).

These results show a relatively poor performance of some standard diagnostic tests and suggest a role for the assessment of oestrogen state in the diagnosis of PCO.

### HOT BIOPSY FORCEPS ARTEFACT IN THE COLON — A CAUSE OF DIAGNOSTIC CONFUSION

**Elizabeth Burd, B. F. Warren, R. A. Mountford, P. Durdey, N. A. Shepherd**
Departments of Pathology, Medicine and Surgery, Bristol Royal Infirmary and Department of Pathology, Gloucestershire Royal Hospital, Gloucester

Small colonic polyps are removed colonoscopically using hot biopsy (diathermy) forceps. This produces considerable distortion in the tissue submitted for histopathological examination and blurs the diagnostic features of normal colonic mucosa, metaplastic polyps and adenomata.

Twenty-five small polyps with varying degrees of hot biopsy forceps artefact were circulated amonst eight pathologists with a range of experience and seniority. Diagnostic and artefactual features were graded on a scale of 0-5. The most useful criteria in assessing the heat damaged areas were surface maturation, overall architecture and comparison of normal with abnormal areas with the same degree of heat damage in the same biopsy.

This study also revealed that thermal artefact can mimic metaplastic features on the surface of normal biopsies and can simulate dysplasia in metaplastic polyps.

Heat damaged tissue from hot biopsy forceps can and should be used in diagnosis, but requires care.

### METASTATIC PROSTATIC CARCINOMA PRESENTING AS LEFT SIDED CERVICAL LYMPHADENOPATHY. A SERIES OF 11 CASES

**H. Jones and P. P. Anthony**
Postgraduate Medical School, University of Exeter.

Eleven cases of metastatic prostatic carcinoma in cervical lymph nodes as a primary presenting sign were identified in a survey of 250 cervical lymph node biopsies from men. The diagnosis was clinically unsuspected in all cases. All occurred on the left side of the neck. These 11 cases represented 36.6% of all metastatic adenocarcinomas in the neck and 52.3% of those with left sided involvement. The diagnosis was readily confirmed by immunostaining for prostatic specific antigen and prostatic specific acid phosphatase. Six patients are alive and well at an average of 25.8 months and five others survived for an average of 34.4 months, the combined survival being 29.7 months. This contrasts with the dismal fate of patients with metastatic adenocarcinoma from other sites who all died at an average of 2 months from diagnosis. Disseminated prostatic carcinoma, whether to non-regional lymph nodes, viscera or skeleton, is treatable with worthwhile results.

### THE NUMB CHIN SYNDROME — A CASE PRESENTATION

**E. L. Blundell**
Department of Haematology
Southmead Hospital, Bristol

A 62 year old lady presented with a 5 week history of lethargy, generalised myalgia, and anorexia with weight loss. With this history and the finding of a raised viscosity (1.84cp) her G.P. had diagnosed polymyalgia rheumatica, but there had been no response to 2 weeks treatment with Prednisolone.

Although clearly unwell, with pallor and global muscular weakness, the only specific finding was loss of sensation over the left lower lip and chin.

She had a leucoerythroblastic blood film (Hb 8.9 g/dl) and normal plasma viscosity. A CT brain scan performed because of progressive neurological deterioration (bilateral upgoing plantars) was normal, and a CT scan of chest and abdomen showed only minimally enlarged para-aortic glands. However, bone marrow aspiration and lumbar puncture showed malignant cells, enabling a diagnosis of high grade B cell lymphoma to be made.

Despite intensive systemic and intrathecal chemotherapy, reassessment at day 14 showed persisting disease. She died from progressive cerebral disease 4 days later.

The Numb Chin Syndrome has been recognised as a possible manifestation of malignant disease frequently haematological. However this has usually been due to compression of the nerve as it passes through the mental foramen, but in this case, histology showed no external compression but infiltration of the nerve by malignant cells.

### THE USE OF TETRAHEDRON AS AN AID TO SPECIMEN RADIOLOGY

**J. S. Armstrong, A. M. Nener, J. D. Davies**
Regional Breast Pathology Unit, Bristol BS10 5NB

Architectural distortions in the breast make up 40% of the impalpable lesions biopsied in the Avon district as a result of breast cancer screening. Such lesions are often difficult to identify on specimen mammograms, since the specimen has been orientated in a different plane from the clinical mammogram. Compression of the specimen aids visualisation of the abnormality, but, specimens may need to be rotated to reveal the lesion. X-raying specimens in three dimensions also reveals information on the size and shape of the lesion in different planes. Hollow tetrahedron shaped containers of varying sizes were constructed from cardboard and coated thin plastic. Specimens were placed in plastic bags and fixed inside the containers. The tetrahedron was then X-rayed on each face. Four X-rays are thus produced, each at an angle of 120° to each other.

Thirty seven consecutive needle localisation biopsies from the UK national breast cancer screening programme were radiographed within the tetrahedron. Examination of specimens in this way aided the identification of the mammographic abnormality. The largest dimension of the lesion could also be selected and the lesion sliced along this plane (as assessed from the four angled specimen mammograms). Alternatively the specimen can be sliced in the same plane as the clinical mammogram to aid radiographic pathological correlation.

This technique is useful both to confirm or exclude excision of the mammographic lesion in difficult cases. This may obviate the need for difficult and painful early post operative clinical mammograms to look for the suspicious lesion in the residual breast tissue. It can also help the pathologist to orientate the specimen and can allow the axis of maximum diameter to be assessed before incision of the lesion.

### SEPSIS SEVERITY SCORING AND ANTIMICROBIAL THERAPY IN ACUTE MENINGITIS DUE TO LISTERIA MONOCYTOGENES

**A MacGowan[2], S. McCulloch[1], J. McLauchlin[3] and D. Reeves**
Departments of Medical Microbiology, Southmead Hospital[1] and Bristol Royal Infirmary[2], Bristol, and Central Public Health Laboratory, London[3]

Previous studies of meningitis due to **L. monocytogenes** have suggested that increasing age, underlying disease, seizures, low CSF glucose and concomitant septicaemia are bad prognostic indicators in adult infection. We reviewed 79 cases of acute meningitis in patients over 18 years occurring between 1986 and 1991 in the British Isles. Coma, seizures, organisms seen on Gram film of the CSF, low platelet count ( 100 x $10^9$/1), WBC of 4.0 or 20.0 x $10^9$/1 and blood urea of 10 mmol/l or creatinine of 150 umol/l were associated with a poor outcome. In contrast there was no significant association between age, underlying disease, focal neurological signs, systolic blood pressure, pulse or bacteraemia and mortality. A sepsis score using a combination of relevant factors could be related to mortality.

Antimicrobial therapy was analysed. Ampicillin or penicillin plus gentamicin was shown to be superior to ampicillin plus chloramphenicol and chloramphenicol alone was superior to ampicillin and chloramphenicol in treatment of meningitis once the aetiology was known. Patients who were treated with chloramphenicol alone or in combination with ampicillin were significantly more likely to require a change in antimicrobials than those treated with ampicillin plus an aminoglycoside. This data confirms and extends previous studies which suggested the unsuitability of chloramphenicol in the treatment of listeria meningitis.

### NSE EXPRESSION IN CARCINOMA OF THE ANAL CANAL: AN IMMUNOHISTOCHEMICAL STUDY

**L. P. Keatings, P. A. Burton**
Department of Histopathology
Southmead Hospital, Bristol

Several cases of primary small cell neuroendocrine carcinoma of the anal canal have been reported in the literature and appear to be highly aggressive neoplasms. These tumours can be shown to contain cytoplasmic neurosecretory granules on electron microscopy. However, at the light microscopic level, small cell neuroendocrine tumours may appear virtually identical to basaloid carcinoma of anus, the principal differential diagnostic alternative.

The purpose of this study was to examine a series of thirty carcinomas of anal canal for evidence of neuroendocrine differentiation using immunohistochemical techniques. A panel of antibodies against NSE, S100 protein, cytokeratins (CAM 5.2) and EMA was employed and the ABC immunoperoxidase method was applied.

The results revealed that 13 out of the 30 tumours stained positively for NSE suggesting that these tumours showed neuroendocrine differentiation. All of the NSE positive tumours were also positive for CAM 5.2 or EMA confirming their epithelial origin. The results with antisera against S100 protein were negative thus excluding melanoma from the differential diagnosis.

These results should perhaps be interpreted with some caution in view of the fact that NSE is known to be expressed by some tumours which are not endocrine or neural in origin. It would be of great interest, however, to correlate the immunostaining with the ultrastructure of the tumours on electron microscopy in order to assess the specificity of the NSE antiserum.

Small cell neuroendocrine carcinoma of the anal canal is a rapidly fatal disease; it would be extremely useful to be able to diagnose these tumours more easily, with the help of immunohistochemical staining and attempt to improve their abysmal prognosis with aggressive treatment regimes.

